# Urticarial Vasculitis Associated With Levothyroxine

**DOI:** 10.7759/cureus.40754

**Published:** 2023-06-21

**Authors:** Mark Metry, Abdel Rahem S Yusuf, Jason Lane, Rashmi Unwala, Roula Altisheh

**Affiliations:** 1 Internal Medicine, Cleveland Clinic Akron General, Akron, USA; 2 Pathology, Cleveland Clinic Akron General, Akron, USA; 3 Dermatology, Cleveland Clinic, Cleveland, USA

**Keywords:** hypothyroidism, hypothyroid, thyroiditis, leukocytoclastic, levothyroxine, vasculitis, urticarial

## Abstract

Urticarial vasculitis (UV) is a protean disorder that can be triggered by a myriad of causes although it is often idiopathic. Treatment is often successful with corticosteroids and/or immunosuppressive drugs. However, when a cause is found, specific treatment of the underlying problem or removal of an offending agent will resolve the symptoms. This report describes a patient with UV triggered by thyroid replacement, necessitated by Hashimoto's thyroiditis, which can itself cause UV. In this unusual presentation, rather than thyroiditis, thyroid replacement was the trigger for the vasculitis.

## Introduction

Many patients with allergic diseases present with cutaneous involvement. Urticarial vasculitis (UV) is a form of cutaneous leukocytoclastic vasculitis that can present with cutaneous and systemic manifestations. UV is more difficult to treat than chronic spontaneous urticaria (CSU) and is associated with the deposition of antigen-antibody complexes in the vascular lumen, sometimes with complement activation and consumption. UV can be limited to the skin or have systemic manifestations, the more severe of which is associated with hypocomplementemia [1] and extracutaneous symptoms such as fever, arthralgia, lymphadenopathy, and renal and pulmonary manifestations [2]. Skin biopsy is essential to make the diagnosis and immunofluorescence studies can detect the deposition of immunoglobulins or complement components in blood vessel walls. Consideration of this diagnosis should be given when approaching a patient with recurrent or treatment-resistant urticarial skin lesions. The objective of this report is to describe a patient who developed long-standing recurrent rashes following administration of levothyroxine, who was subsequently diagnosed with UV. While medication-induced UV does occur, occurrence as a result of levothyroxine administration has not been previously described in the literature.

## Case presentation

**Figure 1:**
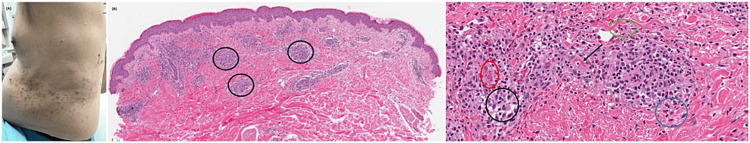
(A) UV rash; (B) low power (5x) photomicrograph showing superficial and mid-reticular dermis with a prominent perivascular inflammatory cell infiltrate; (C) H&E stain at 200x total magnification, showing a brisk perivascular lymphohistiocytic inflammatory infiltrate (black circle) with numerous admixed eosinophils (blue circle). Endothelial cell swelling, mild erythrocyte extravasation (red oval), very focal probable fibrinoid degeneration of blood vessel walls (arrow), and surrounding karyorrhectic debris (green oval) are seen. UV: urticarial vasculitis.

A 68-year-old male with a history of hyperlipidemia, sleep apnea, and hypothyroidism secondary to Hashimoto’s thyroiditis presented with a recurring rash over a period of 12 years. The rash consisted of fixed, non-migratory clusters of wheals and pink papules with hyperpigmented macules along the lower back and lower extremities (Figure 1A). There was no mucosal involvement, joint involvement, or blistering. Symptom onset typically occurred one month after starting any thyroid replacement medication but would resolve within two months of medication cessation and the addition of systemic corticosteroids.

Over a period of several years, the patient tried different thyroid replacement medications with different excipients, starting with Armour Thyroid, branded Synthroid, and Tirosint, under the direction of his endocrinologist. Upon exposure to levothyroxine in each formula, the patient developed the same type of rash a few months into the treatment plan, leading to levothyroxine discontinuation and the use of systemic steroids for resolution. Subsequently, his thyroid replacement was discontinued for four months, but he deteriorated significantly with poor clinical status and was restarted on branded Synthroid. He was advised to try oral antihistamines, which did not improve the course of his rash.

Over a period of seven weeks, he underwent slow oral levothyroxine desensitization initiated at 0.075 mcg daily with weekly increase in doses to reach the target dose of 75 mcg/day for a presumed delayed non-immunoglobulin E (IgE)-mediated hypersensitivity reaction. The patient successfully completed the desensitization protocol; however, his levothyroxine dose remained subtherapeutic. As a result, it was deemed clinically necessary to slowly up-titrate levothyroxine further to 150 µg daily; the rash reoccurred at 88 mcg dosage. Triamcinolone ointments, bleach baths, ivermectin, butenafine, and H1 and H2 blockers did not provide relief from symptoms. He underwent multiple skin biopsies with nonspecific findings until his most recent biopsy, which showed subtle signs of vasculitis (Figure 1B, C).

Direct immunofluorescence (DIF) antibody localization demonstrated negative immunoreactivity for immunoglobulins IgG, IgA, IgM, and complement C3 on sections of frozen skin. UV was suspected, and the diagnosis was confirmed when treatment with 0.6 mg twice daily dosing of colchicine resulted in a good clinical response and subsequent remission of his rash. The patient underwent a thorough evaluation, including, but not limited to, C3 and C4 complement levels, antineutrophil cytoplasmic antibody (ANCA), serum protein electrophoresis (SPEP), hepatitis panel, erythrocyte sedimentation rate (ESR), rheumatoid factor (RF), and cryoglobulin, in search of an underlying cause for his vasculitis, and none was found (Table 1).

**Table 1:** Diagnostic workup lab values. ESR: erythrocyte sedimentation rate; ANCA: antineutrophil cytoplasmic antibody; RF: rheumatoid factor; SPEP: serum protein electrophoresis; CBC: complete blood count; UA: urine analysis; ANA: antinuclear antibody.

Lab	Values
C3	136 (86-166 mg/dL)
C4	17 (13-46 mg/dL)
UA	Unremarkable
ESR	12 (0-15 mm/h)
Hepatitis panel	Negative
ANCA	Negative
RF	11 IU/mL
SPEP	Unremarkable
Immunofixation	No M protein
CBC	Unremarkable
Cryoglobulin	19 ug/mL
ANA	Negative

## Discussion

UV is considered a type III hypersensitivity reaction that may be skin-limited or a systemic disorder; or it may be a feature of another systemic disorder, resulting in the deposition of antigen-antibody complexes in the vascular lumina with subsequent complement activation. It is diagnosed by the presence of both urticaria and histologic evidence of leukocytoclastic vasculitis. UV can be divided into normocomplementemic urticarial vasculitis (NUV), hypocomplementemic urticarial vasculitis (HUV), and a rare hypocomplementemic urticarial vasculitis syndrome (HUVS). Positive DIF findings on skin biopsy are more common in patients with hypocomplementemic forms of UV [3]. While most UV cases are idiopathic, they can be associated with external factors, including drugs or underlying diseases such as thyroiditis or autoimmune diseases.

Many patients with UV have antithyroperoxidase or antithyroglobulin antibodies [4]. In an observational study, a small cohort of patients with CSU and UV were examined, and the authors found antithyroid antibodies more prevalent in the UV population (41.7% in UV compared with 4% in CSU) [5]. Twenty-five to 50% of patients with UV have underlying systemic diseases, including solid organ tumors and hematologic abnormalities, and infectious diseases, including HIV, Lyme, viral hepatitis (A, B, and C), rhinosinusal aspergillosis, systemic lupus erythematosus, and Sjögren’s syndrome [6]. Treatment of the underlying condition usually leads to remission of the UV. The use of immunomodulatory agents, biologics (i.e., anti-tumor necrosis factor (TNF), anti-IgE monoclonal antibody (mAb), and anti-CD20 mAb), often allows corticosteroid tapering and improves the efficacy of therapy [6,7]. When UV is associated with drug reactions, withdrawal of the relevant drug causes remission or improvement of the UV. While biologics reduce the need for long-term systemic steroid use, they also still carry a risk of serious side effects [4,6,7]. In recent years, there have been several case reports highlighting the use of dapsone and colchicine (as well as azathioprine and cyclophosphamide), allowing patients to avoid the need for longer term corticosteroids. Dapsone and colchicine are known to be effective for diseases in which neutrophils play an important role. This patient’s thyroiditis with UV is a known, if uncommon, association [6]. What was unique about his presentation was that his cutaneous lesions were caused by thyroid replacement, rather than being due to his thyroiditis state or some other underlying condition. Through an exhaustive process of elimination as well as long observation over a lengthy course of illness, it became clear there was no other explanation. Due to the clinical necessity for levothyroxine in order to achieve euthyroidism, and with the UV limited to cutaneous symptoms, levothyroxine treatments were continued after carefully weighing the risks and benefits of such an approach in a shared medical decision-making fashion with a plan for careful monitoring. 

## Conclusions

Nonetheless, this case illustrates the importance of keeping UV in the differential diagnosis for patients with thyroid disease who present with atypical wheals. To the best of our knowledge, this is the first case demonstrating UV associated with the administration of levothyroxine.
